# Persisters: a distinct physiological state of *E. coli*

**DOI:** 10.1186/1471-2180-6-53

**Published:** 2006-06-12

**Authors:** Devang Shah, Zhigang Zhang, Arkady Khodursky, Niilo Kaldalu, Kristi Kurg, Kim Lewis

**Affiliations:** 1 Department of Biology, Northeastern University, 134 Mugar Hall, 360 Huntington Ave, Boston, MA 02115, USA; 2 Department of Chemical Engineering and Materials Science, University of Minnesota, St Paul, MN 55108, USA; 3 BioTechnology Institute, University of Minnesota, St Paul, MN 55108, USA; 4 Department of Biochemistry, Molecular Biology and Biophysics, University of Minnesota, St Paul, MN 55108, USA; 5 Institute of Technology, Tartu University, Tartu 50411, Estonia

## Abstract

**Background:**

Bacterial populations contain persisters, phenotypic variants that constitute approximately 1% of cells in stationary phase and biofilm cultures. Multidrug tolerance of persisters is largely responsible for the inability of antibiotics to completely eradicate infections. Recent progress in understanding persisters is encouraging, but the main obstacle in understanding their nature was our inability to isolate these elusive cells from a wild-type population since their discovery in 1944.

**Results:**

We hypothesized that persisters are dormant cells with a low level of translation, and used this to physically sort dim *E. coli *cells which do not contain sufficient amounts of unstable GFP expressed from a promoter whose activity depends on the growth rate. The dim cells were tolerant to antibiotics and exhibited a gene expression profile distinctly different from those observed for cells in exponential or stationary phases. Genes coding for toxin-antitoxin module proteins were expressed in persisters and are likely contributors to this condition.

**Conclusion:**

We report a method for persister isolation and conclude that these cells represent a distinct state of bacterial physiology.

## Background

Persisters are multidrug tolerant cells present in all bacterial populations studied to date [1]. Persisters are not mutants, but rather phenotypic variants of the wild-type that upon reinoculation produce a culture with similar levels of tolerance [2-4]. The number of persisters in *Escherichia coli *(*E. coli*) remains constant throughout early-exponential phase, with a marked increase as cells enter late-exponential and early-stationary phases [3]. Maintaining cells in exponential growth using repeated dilutions in fresh media, similar to growth in a chemostat, resulted in a complete loss of persisters [3]. This lack of persistence demonstrates that these cells are not at a particular stage in the cell cycle as originally suggested by Moyed [5], and are not produced in response to antibiotics. In a recent study employing a microfluidic device to monitor cell growth, persisters were shown to be rare non-growing cells that pre-exist in a population [10]. Persisters are responsible for multidrug tolerance of biofilms [1] which account for the majority of infectious diseases in the developed world [6,7].

We previously reported isolation of persisters from a culture of an *E. coli hipA7 *(high persistence) mutant [5] that was lysed with ampicillin [8]. Intact persisters were collected and their gene expression profile indicated overexpression of chromosomal toxin-antitoxin (TA) modules. "Toxins" cause reversible stasis by blocking essential functions, such as translation [9], and appeared as promising candidates for MDT genes. Overexpression of RelE or HipA toxins caused a sharp increase in persistence, while deletion of the *hipBA *module strongly decreased the number of persisters in both stationary and biofilm cultures. The same *hipBA *deletion mutant exhibited no change in persistence during exponential growth or when grown in minimal media, suggesting that persister formation is governed by redundant genes whose activity depends on particular conditions (indeed, there are >10 TA modules in *E. coli *[11]). Recent progress in understanding persisters is encouraging, but the main obstacle in understanding their nature was our inability to isolate these elusive cells from a wild-type population without antibiotic treatment since their discovery in 1944 [2].

We reasoned that the apparent dormancy of persisters [10] could be exploited to physically isolate these cells. A strain expressing degradable GFP from a ribosomal promoter that is only active under conditions of rapid growth was used to physically sort dim persister cells from the bulk of the population. Here, we report a method of isolating naive persisters from wild-type *E. coli *and based on their gene expression profile conclude that they represent a third physiological state of bacterial cells, distinct from both exponential and stationary forms.

## Results and discussion

**Figure 1:**
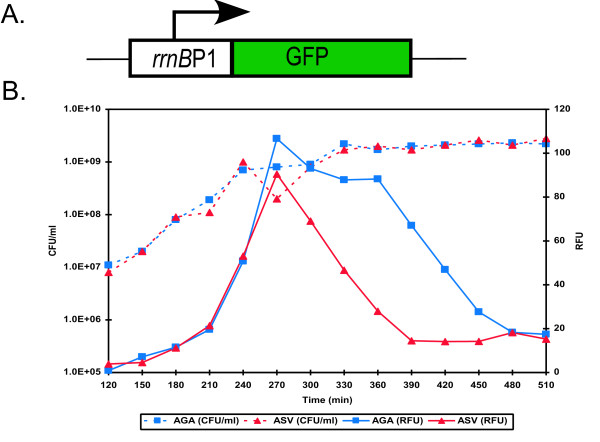
**Degradable GFP expression from a ribosomal promoter in a growing culture of *E. coli***. (A) Graphical representation of the reporter. An unstable variant of GFP was placed downstream of a ribosomal promoter, *rrnB*P1. (B) Stationary phase cultures of *E. coli *ASV and AGA, each containing a different variant of unstable GFP, were diluted (1:1000) in fresh media and cultured with aeration at 37°C. At designated timepoints samples were removed and assayed for cell counts (CFU/ml) and fluorescence (RFU, arbitrary units).

Dormant cells are expected to have a low rate of protein synthesis, thereby providing the basic rationale for sorting persisters from the population. *E. coli *strains ASV and AGA carry gene cassettes encoding previously described [12] unstable variants of GFP under the control of the ribosomal *rrnB*P1 promoter (Fig. 1A). These gene cassettes are inserted as a single copy into the λ phage attachment site of the chromosome (Søren Molin, unpublished). *rrnb*P1 normally controls expression of the *rrnB *gene which codes for 16S rRNA and is expressed at high levels during growth [13,14]. The half-life of this unstable GFP is <1 hour, and it is rapidly cleared from non-growing cells [12]. In a growing culture, fluorescence was bright at exponential state, and subsequently lost shortly after cells entered stationary state (Fig. 1B). This provided a unique opportunity to sort bright and dim cells and analyze their physiology.

**Figure 2:**
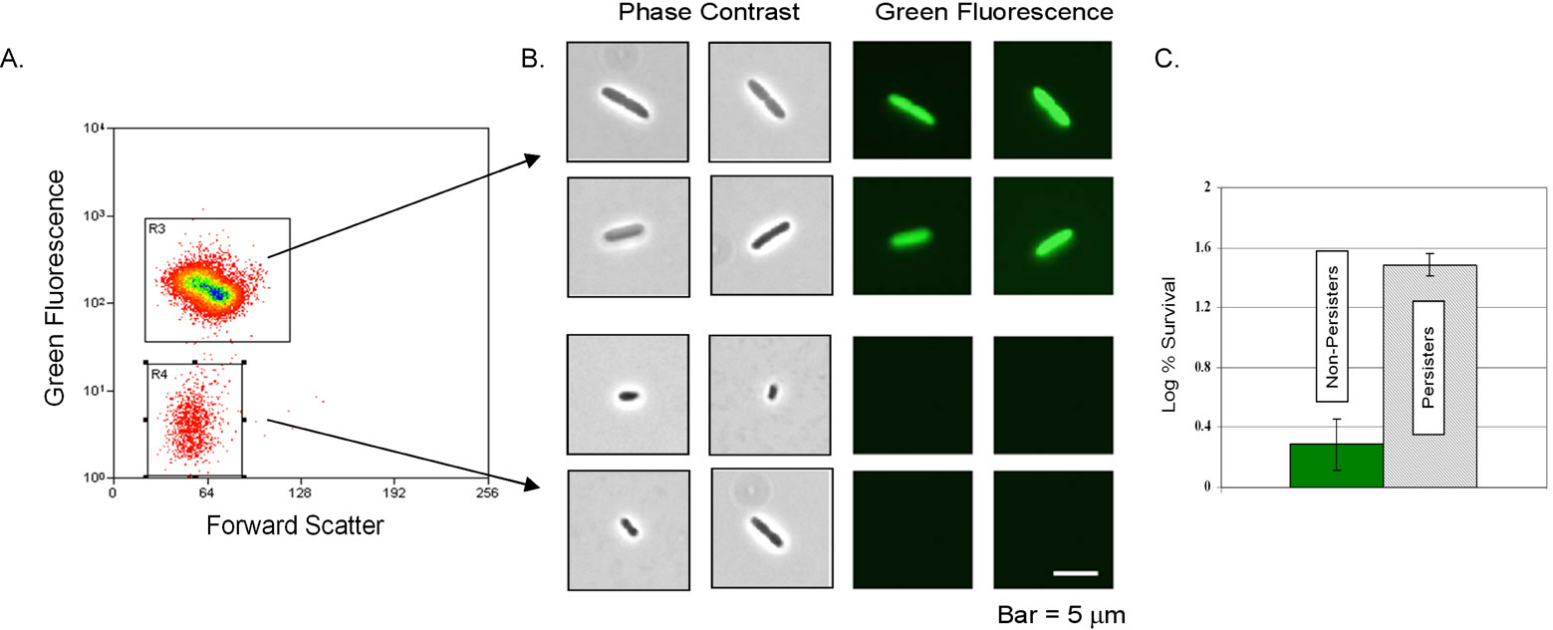
**Isolation of persister cells from an exponentially growing culture**. *E. coli *ASV cells containing this reporter cassette were grown in LB medium to mid-exponential phase (~1 × 10^8 ^cells/ml) at 37°C with aeration and sorted using a high speed cell-sorter equipped with a standard GFP filter set. (A) Two populations were detected using forward light-scatter, one that fluoresced brightly (R3), and another that did not (R4). (B) The sorted populations were visualized by epifluorescent microscopy (bar, 5 μm). (C) Cells were sorted as described in (A-B). Once sorted both populations were treated with ofloxacin (5 μg/ml) for three hours, diluted and spotted onto LB agar plates for colony counts.

An exponentially growing population of *E. coli *ASV (cultured for one hour to a cell density of approximately 10^8 ^CFU/ml) was sorted with a MoFlo cell-sorter using forward light scatter, which allows detection of particles based on size. This enabled detection of cells irrespective of their level of fluorescence. Fluorescence of GFP in individual cells was recorded simultaneously using laser excitation and light detection. Fluorescence activated cell sorting (FACS) analysis showed that the population consisted of two strikingly different types of cells – a bright majority, and a small subpopulation of cells with no detectable fluorescence (Fig. 2A). The two populations were sorted based on fluorescent intensity and collected in phosphate buffer. Epifluorescent microscopy confirmed that the sorted bright cells were indeed bright green, while the dim ones had no detectable fluorescence (Fig. 2B). The dim cells were also smaller than the fluorescent cells, and in this regard resembled stationary state cells.

Sorting was performed in a non-nutritive buffer to prevent persisters from reverting back into growing cells. Therefore, under the sorting conditions, regular cells stopped growing. This limited the choice of antibiotics we could use to probe for tolerance to fluoroquinolones, which have the ability to kill normal non-growing cells [15]. Sorted cells were exposed to a high level of a bactericidal antibiotic ofloxacin (an inhibitor of DNA gyrase) which rapidly kills both growing and non-growing normal cells, but has no effect on persisters [15,3]. The dim subpopulation had a 20-fold higher survival rate as compared to the sorted bright cells (Fig. 2C). This experiment demonstrates that the sorted dim population was in fact enriched for cells exhibiting a persistent phenotype. This result also validates the hypothesis that these persisters are dormant cells with low levels of protein synthesis. Importantly, the dim cells were sorted out from a population of wild-type *E. coli *that was not pre-treated with an antibiotic. We therefore conclude that persisters are dormant cells that are formed within a normally distributed population.

The ability to sort and purify a population of persisters presented an opportunity to examine their gene expression profile. In order to collect sufficient numbers of persisters, several cultures of ASV were inoculated independently at 30 minute intervals, and persisters were isolated on two MoFlo instruments running in parallel, with cells harvested in each case at the same time point during growth. Once sorted, cells were maintained on ice for several hours until all were collected. In order to concentrate the highly dilute suspensions, cells were co-precipitated by centrifugation with polystyrene beads.

**Figure 3:**
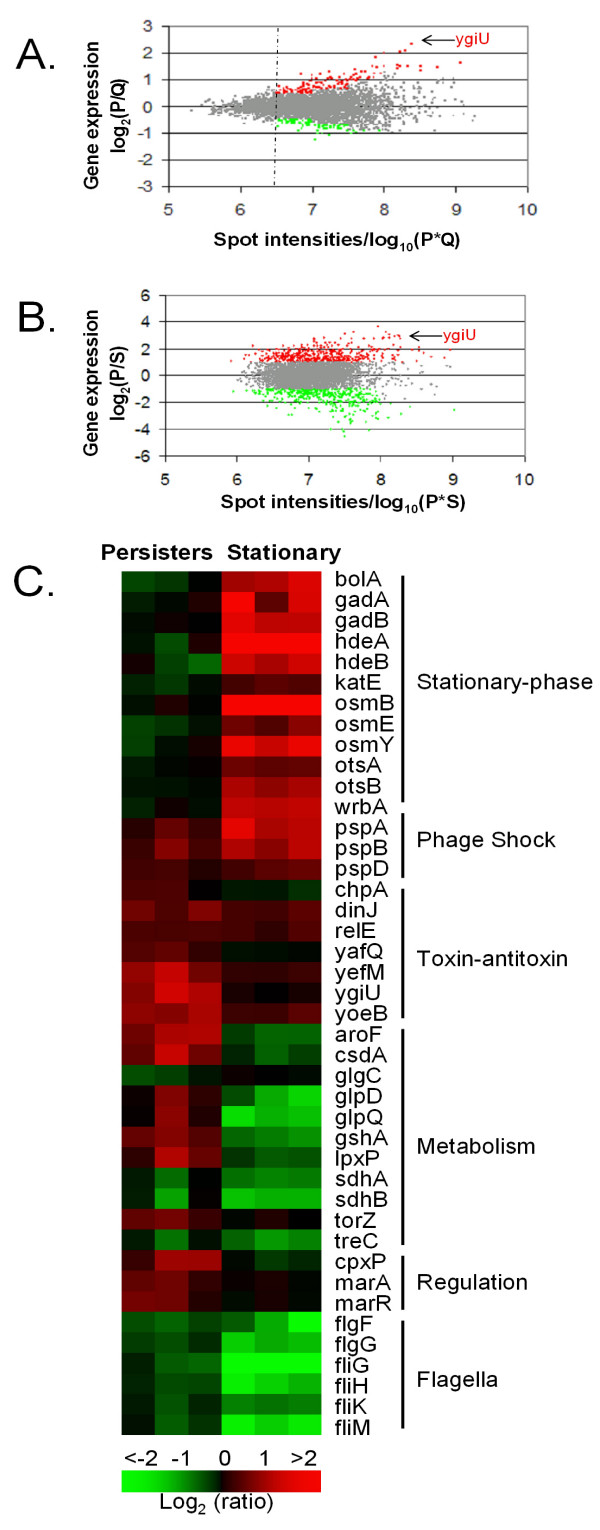
**Gene expression profile of FACS isolated *E. coli *persisters**. *E. coli *ASV Cells were sorted as described in legend, Fig. 2. cDNA was prepared from the total RNA and hybridized to spotted *E. coli *DNA microarrays [16]. Data shown are the averages of three independent biological replicates. Genes up-regulated in persisters are indicated as red and those genes which are repressed as green. The differential expressed genes are identified according to the procedures described in *Materials and Methods*. (A) Gene expression and spot intensities of sorted persisters (*P*) compared to sorted non-persisters (*Q*) from an exponential growth phase cell culture. (B) Gene expression and spot intensities of sorted persisters (*P*) compared to stationary phase (*S*) cells. (C) Heat map comparison of representative genes differentially expressed in persisters *(P) *or stationary *(S) *phase cells as compared to non-persisters *(Q) *and exponential growth phase cells *(L)*, respectively. Genes are considered differentially expressed if the local intensity-dependent Z-score is greater than 1.96 or less than -1.96 and the expression log2 (ratio) is more than 1 or less than -1. The gene names are shown on the right, together with the functional groups.

For genome-wide expression profiling, total RNA was purified from sorted dim and bright cells in three independent experiments. cDNA was prepared from total RNA and hybridized to spotted microarrays representing ~4,400 open reading frames (ORF's) of *E. coli *[16]. Approximately 5% of the genes in persisters showed statistically significant differential expression, identified as described in *Materials and Methods*, when compared to the sorted non-persister cells (Fig. 3A). 45 genes showed at least a two-fold increase in expression, (Fig. 3A), while 5 genes were significantly down-regulated in persisters (see Additional file 1).

When compared to stationary phase cells, persisters also showed significant differences in gene expression (Fig. 3B). Nearly ~420 genes are up-regulated in persisters, while roughly the same number of genes was down-regulated (Fig. 3B). Unexpectedly, persisters appear more similar to exponential, than stationary phase cells.

The gene profile of persisters as compared to exponentially dividing cells showed down-regulation of genes involved in energy production and non-essential functions such as flagellar synthesis, consistent with a dormant state (Fig. 3C). Expression of flagellar genes was particularly strongly suppressed, indicating that persistence is the opposite of an actively mobile state.

The unique persister transcriptome pointed to genes that were likely to contribute to dormancy. These were the elements of the "toxin-antitoxin" (TA) modules *dinJ*, *yoeB*, and *yefM*. Our previous studies, where persisters were isolated by lysing a population of *hipA7 *mutants of *E. coli *with ampicillin, also indicated overexpression of TA modules, but apart from *dinJ*, the prominent overexpressed genes were *relE *and *MazF*, rather than *yefM *and *yoeB *[8]. It is possible that *hipA7 *persisters are similar, but not identical to the ones formed by wild-type cells, although the general principle, and perhaps the overall mechanism of their formation, appears to be the same. "Toxins" [17] would be uniquely well suited for initiating cell dormancy. RelE and MazF are mRNA endonucleases that inhibit translation [18-20] and can cause reversible stasis [9]. YoeB expressed in wild-type persisters is a RelE homolog, and YefM is its antitoxin. DinJ is the cognate antitoxin of another RelE homolog, YafQ. The gene most highly expressed in persisters as compared to non-persisters was *ygiU*. Based on sequence similarity, *ygiU *was predicted to be a cyanide hydratase and also shown to be induced upon biofilm formation [21,22]. More recently, *ygiU *has been described as a global regulator that controls biofilm formation by inducing motility via the two-component regulatory system QseBC [23]. It is unclear how this may relate to persistence but it is also interesting that *ygiU *is part of a two-gene operon along with *ygiT*, which is annotated as a transcriptional repressor. In this regard *ygiUT *resembles a TA module, in particular *higAB*, a well characterized TA module where the toxin is also located upstream of the antitoxin [24,25]. Note that atitoxins typically act as transcriptional repressors of their operons. A null mutant of *ygiT *cannot be obtained, further suggesting that *ygiUT *may also function as a previously undescribed TA pair (Niilo Kaldalu, unpublished).

**Figure 4:**
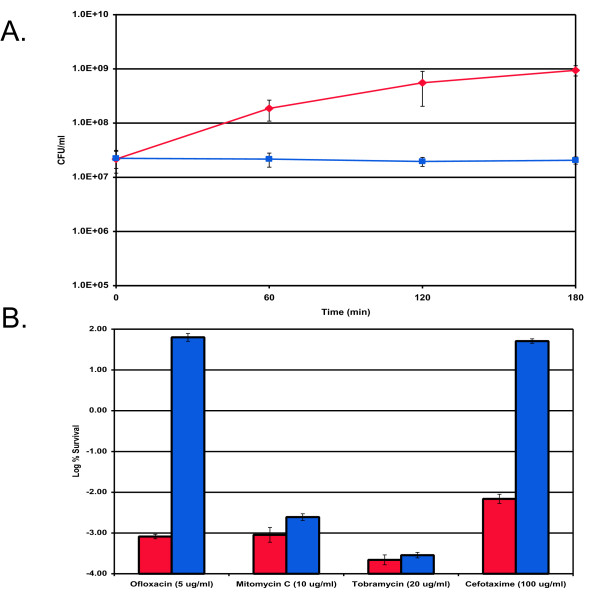
**Effects of *ygiU *overexpression on persister formation**. *E. coli *MG1655 cells were grown in LB medium to mid-exponential phase (~5 × 10^7 ^cells/ml) at 37°C with aeration. (A) *ygiU *expression was induced (squares) from pTOX at T = 0 by the addition of 1 mM arabinose, and MG1655 with a blank vector (pBAD33) served as the control (diamonds). (B) Cells were cultured, and *ygiU *expression was induced as described in (A). After 2 h of *ygiU *induction, samples were removed and treated with either cefotaxime (100 μg/ml), mitomycin C (10 μg/ml), ofloxacin (5 μg/ml), or tobramycin (20 μg/ml) for 3 h at 37°C with aeration. The control (MG1655/pBAD) (red bars) was challenged at a cell density similar to that of the *ygiU *induced cells (blue bars).

It was previously shown that overexpression of RelE [8] or HipA [26] can cause a dormant, multidrug tolerant state. In view of the findings described here, it was of interest to determine whether YgiU had the capability of inducing persister formation as well. Attempts to clone *ygiU *into an expression vector pTOX under the control of a tight arabinose promoter were unsuccessful, apparently due to the small amount of leakage from the promoter. Therefore, *ygiU *was then cloned into a strain carrying the antitoxin, *ygiT*, under an IPTG-inducible promoter on the pATOX-2 expression vector. A strain of *E. coli*, MG1655, containing both plasmids was cultured to mid-exponential phase and YgiU production was induced by arabinose. Growth ceased very quickly after the addition of arabinose, and remained inhibited for the duration of the experiment (Fig. 4A). To test for tolerance, samples were removed 120 min after arabinose induction, and exposed to high concentrations of bactericidal antibiotics [3]. The antibiotic panel included ofloxacin, cefotaxime, mitomycin C, and tobramycin. A dramatic increase in tolerance (10,000–100,000 fold) to ofloxacin and cefotaxime, a β-lactam antibiotic, was observed in cells overexpressing YgiU (Fig. 4B). This result correlates well with the tolerance of isolated persisters to ofloxacin and a different β-lactam, ampicillin, described above. Complete tolerance to cefotaxime was expected, since β-lactams are ineffective against non-growing cells. Complete protection from ofloxacin is unique – expression of other toxins, such as RelE or HipA protects cells from fluoroquinolones, but not to the same extent [8]. YgiU did not protect cells from tobramycin, an aminoglycoside inhibitor of translation, or from mitomycin, which forms DNA adducts. In contrast, RelE was previously observed to protect cells from aminoglycosides, and HipA caused tolerance to all antibiotics tested [8]. It appears that the action of YgiU is strong and selective, pointing to a possible interaction with DNA gyrase or topoisomerase IV, the targets of fluoroquinolones. Indeed known toxins CcdB and ParE act by inhibiting DNA gyrases [11]. Another interesting example of a protein specifically protecting cells from fluoroquinolones is QnrA [27,28], and it was recently reported that its *M. tuberculosis *homolog, MfpA acts by directly binding the topoisomerase [29]. The sequestered enzyme can no longer cleave the DNA in the presence of fluoroquinolones.

A knockout of *ygiU*, or *yoeB*, or both, had no effect on persister formation (not shown). This is similar to our previous findings of the lack of a phenotype in knockouts of *relE *or *mazF *genes, the overexpression of which induces tolerance. HipA so far is the only toxin whose elimination decreases the occurrence of persisters, but only under certain conditions (rich medium, stationary phase) [8]. This suggests that persister genes are redundant, and multiple knockouts would be required in most cases to observe a phenotype. Indeed, in *E. coli *the number of TA modules that could contribute to dormancy is >10 [30]. The number of TA modules in *M. tuberculosis *that forms a dormant, persistent carrier state is >60 [31,32].

Antitoxins have been found to act as repressors of TA modules [17], and are susceptible to proteolysis. A decrease in antitoxin protein level causes an induction in transcription, which we observed for some of the antitoxins in the gene profile of persisters. *ygiT *was not differentially expressed in persisters. It is possible that much of the regulation happens at the protein level, transcription profiling is limited in its ability to reflect all TA protein changes, and as a result is likely to miss some persister genes. *hipBA *is a case in point – we did not observe transcriptional overexpression of this element, while a strong phenotype of the Δ*hipBA *strain suggests its importance in persister formation. Future studies are being designed to track protein levels in persisters, such as toxin/antitoxin ratios, and we are currently working on obtaining a persister proteome.

The expression profile of persisters is very different from that of non-growing stationary phase cells (Fig. 3B). We do not see the characteristic stationary phase genes such as *bolA, katE *or *osmB *expressed in isolated persisters. Conversely, TA modules are not highly expressed in stationary phase (Fig. 3C). This shows that persisters differ from both exponentially growing and stationary cells, and constitute a distinct physiological state.

**Figure 5:**
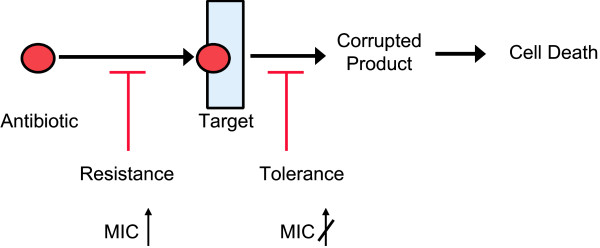
**Multidrug resistance vs. Multidrug tolerance**. Antibiotics normally kill cells by corrupting a particular target or function, ultimately leading to cell death. Resistance mechanisms function by preventing an antibiotic from binding to its target. This allows cells to grow in the presence of an elevated concentration of antibiotic, thereby increasing the MIC. It is proposed that the mechanism of tolerance is based on the non-lethal inhibition of antibiotic targets by specific MDT proteins. If this is the case, blocking of essential targets of antibiotics will produce a partially dormant, multidrug tolerant cell without increasing the MIC.

We have a relatively good understanding of drug resistance mechanisms, but the nature of persister multidrug tolerance (MDT) has remained largely unknown. All resistance mechanisms function by preventing an antibiotic from binding to its target (Fig. 5). This allows cells to grow in the presence of an elevated concentration of antibiotic and increases its minimal inhibitory concentration (MIC). We suggested that the mechanism of tolerance was based on the non-lethal inhibition of antibiotic targets by specific MDT proteins [8]. For example, aminoglycoside antibiotics kill the cell by interrupting translation and producing toxic misfolded peptides; inhibition of translation by an MDT protein would not interfere with aminoglycoside binding, but would prevent killing. Other antibiotics kill the cell by similarly corrupting (rather than merely inhibiting) the target function and it is possible that inhibition of these targets will also prevent killing without increasing the MIC (Fig. 5). If this is the case, blocking of essential targets of antibiotics will produce a partially dormant, multidrug tolerant cell.

The multitude of TA modules that can induce multidrug tolerance is reminiscent of the many MDR pumps responsible for multidrug resistance. *P. aeruginosa*, for example, contains genes coding for 15 MDR pumps belonging to the resistance-nodulation-cell division (RND) family alone, of which a single one, MexAb-OprM, is expressed at a high level under laboratory conditions [33]. Knockouts of most MDR genes produce no phenotype, while overexpression produces a functional MDR pump [34]. It appears that microbial populations have evolved two complementary and highly redundant strategies to protect themselves from antimicrobials – multidrug efflux; and when this fails, multidrug tolerance of persister cells.

## Conclusion

Here, we present a method for isolating naive persisters from wild-type *E. coli*. The method relies on the hypothesis that persisters are dormant cells with a low level of translation and could be applied to all types of bacteria. Genes coding for toxin-antitoxin module proteins, including a novel and previously unidentified toxin, were expressed in persisters and are likely contributors to this condition. Based on their unique gene expression profile we conclude that persisters represent a third physiological state of bacterial cells, distinct from both exponential and stationary forms.

## Methods

### Bacterial strains and growth conditions

Luria-Bertani (LB) broth and LB agar media were used for culturing. Unless indicated otherwise, cells were grown by dilution of overnight cultures 1:1,000 in 12 to 25 ml of LB and incubation in 125-ml baffled culture flasks (Belco) for 2.5 h with aeration (250 rpm) at 37°C. Overnight cultures were made by dilution of thawed cells from an 8% dimethyl sulfoxide stock (-80°C) and incubation in LB medium with aeration for 16 to 20 h. For persister sorting experiments, *E. coli *MG1655-ASV(ASV) was diluted 1:100 in two culture tubes (17 by 100 mm), each containing 1.5 ml of LB broth for a total of 3 ml for each replicate, and incubated at 37°C with aeration for 1 h prior to sorting. For stationary phase experiments, strains were cultured for 16 to 18 h, thereby reaching stationary state prior to testing. For toxin induction and protection studies, cells were cultured in LB containing 100 μg of chloramphenicol/ml and 100 μg of ampicillin/ml, and inducers were added at appropriate times.

### Strain and plasmid construction

Gene deletions were transduced into MG1655 using bacteriophage P1 from an ordered library of deletion mutants that were created replacing corresponding ORF's with a gene coding for resistance to kanamycin [35]. To create double-deletion mutants the Km^r ^cassette was removed by transformation of cells with pCP20 and selection of ampicillin-resistant colonies at 30°C. Colonies were then purified by reinoculation and growth at 43°C. At the end of the procedure, the selected colonies were tested on ampicillin and kanamycin plates to verify the loss of all selective markers. Plasmids pTOX and pATOX-2 were constructed as follows. Briefly, pTOX was constructed by amplifying *ygiU *using primers *ygiU*P1, GGGGTACCTAAGGAGATATATGGAATAATGGAAAAACGCACACCACA and *ygiU*P2, ACATGCATGCTTACTTCTCCTTAAACGAGA and cloning it into the Kpn I and Sph I site of pBAD33[36]. pATOX-2 was constructed by amplifying *ygiT *using primers *ygiT*P1, CGGGGTACCTAAGGAGATATATGGAATAATGAAATGTCCGGTTTGCCA and *ygiT*P2, CCGGAATTCTTAACGGATTTCATTCAATA and cloning it into Kpn I and EcoR I site of pBRlacItac [37].

### ASV Growth/fluorescence assay

*E. coli *ASV and AGA were diluted 1:1000 in culture tubes (17 by 100 mm), each containing 1.5 ml of LB broth, and incubated at 37°C for ~8 hrs. At designated time points samples were withdrawn, diluted in LB medium and spotted on an LB agar plate for colony counts. Additionally, 200 μL of culture was transferred to a black 96-well flat-bottom microtiter plate (NUNC) and fluorescence was measured with a Spectramax Gemini XS spectrofluorometer (Molecular Devices) at a 475-nm excitation wavelength and a 515-nm emission wavelength.

### Persister sorting

*E. coli *ASV was diluted 1:100 in culture tubes (17 by 100 mm), each containing 1.5 ml of LB broth, and incubated at 37°C for 1 h prior to sorting. After 1 h cultures were sorted using a MoFlo (DakoCytomation) cell sorter equipped with a 488 nm laser running at 100 mW. A log scale was used for all parameters measured including side-scatter (SSC), forward-scatter (FSC) and green fluorescence (FL1), which was detected using standard GFP filter sets. Cells were sorted directly into 50 ml centrifuge tubes that contained 3 ml phosphate buffered saline (PBS) with 1 mg/ml bovine serum albumin (BSA). Static charge build-up was dissipated by attaching grounding wires to the instrument and keeping them in contact with the buffer in collection tubes. Once sorted, 100 μl aliquots were removed from the tubes for antibiotic susceptibility measurements. The remaining cell suspensions were kept on ice for several hours until they could be pooled and concentrated. Concentration of cell suspensions was accomplished by mixing and co-sedimenting cells with polystyrene beads ranging from 3–5 μM in diameter (Spherotech, Inc.)

### Antibiotic susceptibility measurements

100 μl aliquots of sorted cells were dispensed in culture tubes (17 by 100 mm) and ofloxacin was added at a final concentration of 5 μg/ml. The tubes were left at room temperature for 3 hours at which point samples were withdrawn, diluted in PBS and spotted on an LB agar plate for colony counts.

### Epifluorescent microscopy

Cells were viewed with an epifluorescence microscope (Zeiss Axioskop 2 plus) with the appropriate filter sets. Images were captured with an Axiocam HRC and associated software (Carl Zeiss, Inc.)

### DNA microarray analysis

Total RNA from stationary (S) and exponential phase (L) cells as well as FACS isolated persisters (P) and non-persisters (Q) was purified using the Qiagen RNeasy kits (Chatsworth, CA) according to the manufacturer's protocols. To identify persister-specific gene expression profiles, relative mRNA levels were determined by parallel two-color hybridization at single-gene resolution to whole-genome *E. coli *K-12 MG1655 spotted DNA microarrays, designed, printed and probed as described [38,16,39], and containing discrete sequence elements corresponding to 98.8% of all annotated open reading frames (ORF's). Complementary DNA probes were synthesized using 0.5 μg of total RNA for sorted cells and 15 μg of nonsorted exponential and stationary phase cells with random hexamers and Cy-5-dUTP or Cy-3 dUTPdyes (Amersham), following hybridization and washing as described previously [16]. The microarray slides were air dried by brief centrifugation and scanned with an Axon Genepix 4000B laser scanner at 10 μm per pixel resolution. The resulting 16-bit TIFF images were analyzed by using the software Genepix Pro 3.0 (Axon). The fluorescence intensity data were first normalized globally using an iterative mean-log_2_(ratio)-centering approach. Fluorescence intensity dependent effects in log_2_(ratio) values were removed by using locally weighted linear regression (lowess) procedure [40]. The normalized Cy-5/Cy-3 ratio for the median was taken to reflect the relative gene expression level changes. The gene functions were obtained from GenProtEc database [41].

Microarray hybridization from 0.5 μg of total RNA yielded the similar image qualities as starting from conventional 15 μg of total RNA, as indicated by Boccazzi *et al*. [42]. Spots signal-to-noise ratios were routinely greater than five from both channels. The experimental error of the RNA abundances was assessed from at least three independent replicates. Each replicate RNA sample corresponds to cells isolated from at least two FACS isolation experiments. The differentially expressed genes were identified using an intensity-dependent *Z*-score threshold method described previously[40,43]. In brief, in an ratio-intensity (R-I) plot, using a sliding window of fixed width in log_10_(Cy5_*_Cy3), the local mean and standard deviation in terms of log_2_(Cy5/Cy3) were calculated within the window surrounding each data point. The *Z*-score simply measures the number of standard deviation of a particular array element *i *is from the mean, defined as

Zilocal=log⁡2(Cy5Cy3)σlog⁡2(Cy5Cy3)local
 MathType@MTEF@5@5@+=feaafiart1ev1aaatCvAUfKttLearuWrP9MDH5MBPbIqV92AaeXatLxBI9gBaebbnrfifHhDYfgasaacH8akY=wiFfYdH8Gipec8Eeeu0xXdbba9frFj0=OqFfea0dXdd9vqai=hGuQ8kuc9pgc9s8qqaq=dirpe0xb9q8qiLsFr0=vr0=vr0dc8meaabaqaciaacaGaaeqabaqabeGadaaakeaacqWGAbGwdaqhaaWcbaGaemyAaKgabaGaemiBaWMaem4Ba8Maem4yamMaemyyaeMaemiBaWgaaOGaeyypa0ZaaSaaaeaacyGGSbaBcqGGVbWBcqGGNbWzdaWgaaWcbaGaeGOmaidabeaakiabcIcaOmaalaaabaGaem4qamKaemyEaKNaeGynaudabaGaem4qamKaemyEaKNaeG4mamdaaiabcMcaPaqaaiabeo8aZnaaDaaaleaacyGGSbaBcqGGVbWBcqGGNbWzdaWgaaadbaGaeGOmaidabeaaliabcIcaOmaalaaabaGaem4qamKaemyEaKNaeGynaudabaGaem4qamKaemyEaKNaeG4mamdaaiabcMcaPaqaaiabdYgaSjabd+gaVjabdogaJjabdggaHjabdYgaSbaaaaaaaa@5C19@

The differentially expressed genes at the 95% confidence level would be those with a value of |Zilocal
 MathType@MTEF@5@5@+=feaafiart1ev1aaatCvAUfKttLearuWrP9MDH5MBPbIqV92AaeXatLxBI9gBaebbnrfifHhDYfgasaacH8akY=wiFfYdH8Gipec8Eeeu0xXdbba9frFj0=OqFfea0dXdd9vqai=hGuQ8kuc9pgc9s8qqaq=dirpe0xb9q8qiLsFr0=vr0=vr0dc8meaabaqaciaacaGaaeqabaqabeGadaaakeaacqWGAbGwdaqhaaWcbaGaemyAaKgabaGaemiBaWMaem4Ba8Maem4yamMaemyyaeMaemiBaWgaaaaa@3634@| > 1.96. The *P*-value for the paired student's *t*-test according to normalized fluorescence intensities for each gene was also calculated. The identified differentially expressed routinely have a *P*-value less than 0.1.

### Toxin induction

Toxin synthesis from the recombinant expression vector was induced by adding L-arabinose at a final concentration of 1 mM to each flask. At the designated time points, a sample was removed, diluted, and spotted on LB agar plates.

### Toxin protection assay

Toxin synthesis was induced by adding L-arabinose at a final concentration of 1 mM to each flask. At the designated time points, a 1-ml sample was removed from each flask and placed in culture tubes (17 by 100 mm) with the appropriate drug concentration, and the tubes were incubated (250 rpm) at 37°C for 3 h. After the antibiotic challenge, cells were washed once (10,000 × g for 1 min) with fresh medium to minimize antibiotic carryover effects, diluted, and spotted onto an LB agar plate for cell counting. A control sample (prior to the addition of antibiotic) was spotted as well.

## Authors' contributions

DS carried out the cell sorting, isolation of total RNA and molecular genetic assays. AK and ZZ conducted the microarray experiments and data analysis. NK and KK designed and tested toxin/antitoxin constructs. KL, DS, ZZ, and NK drafted the manuscript. KL and DS conceived of the study, and participated in its design and coordination. All authors read and approved the final manuscript.

## Supplementary Material

Additional File 1 Excel file containing all gene expression data. Click here for file

## References

[B1] Lewis K (2001). Riddle of biofilm resistance. Antimicrob Agents Chemother.

[B2] Keren I, Kaldalu N, Spoering A, Wang Y, Lewis K (2004). Persister cells and tolerance to antimicrobials. FEMS Microbiol Lett.

[B3] Wiuff C, Zappala RM, Regoes RR, Garner KN, Baquero F, Levin BR (2005). Phenotypic tolerance: antibiotic enrichment of noninherited resistance in bacterial populations. Antimicrob Agents Chemother.

[B4] Bigger JW (1944). Treatment of staphylococcal infections with penicillin.. Lancet.

[B5] Moyed HS, Bertrand KP (1983). hipA, a newly recognized gene of Escherichia coli K-12 that affects frequency of persistence after inhibition of murein synthesis. J Bacteriol.

[B6] Balaban NQ, Merrin J, Chait R, Kowalik L, Leibler S (2004). Bacterial persistence as a phenotypic switch. Science.

[B7] Costerton JW, Stewart PS, Greenberg EP (1999). Bacterial biofilms: A common cause of persistent infections. Science.

[B8] Stewart PS (2002). Mechanisms of antibiotic resistance in bacterial biofilms. Int J Med Microbiol.

[B9] Keren I, Shah D, Spoering A, Kaldalu N, Lewis K (2004). Specialized persister cells and the mechanism of multidrug tolerance in Escherichia coli. J Bacteriol.

[B10] Pedersen K, Christensen SK, Gerdes K (2002). Rapid induction and reversal of a bacteriostatic condition by controlled expression of toxins and antitoxins. Mol Microbiol.

[B11] Gerdes K, Christensen SK, Lobner-Olesen A (2005). Prokaryotic toxin-antitoxin stress response loci. Nat Rev Microbiol.

[B12] Sternberg C, Christensen BB, Johansen T, Toftgaard Nielsen A, Andersen JB, Givskov M, Molin S (1999). Distribution of bacterial growth activity in flow-chamber biofilms. Appl Environ Microbiol.

[B13] Gourse RL, de Boer HA, Nomura M (1986). DNA determinants of rRNA synthesis in E. coli: growth rate dependent regulation, feedback inhibition, upstream activation, antitermination. Cell.

[B14] Bartlett MS, Gourse RL (1994). Growth rate-dependent control of the rrnB P1 core promoter in Escherichia coli. J Bacteriol.

[B15] Spoering AL, Lewis K (2001). Biofilms and planktonic cells of Pseudomonas aeruginosa have similar resistance to killing by antimicrobials. J Bacteriol.

[B16] Khodursky AB, Bernstein JA, Peter BJ, Rhodius V, Wendisch VF, Zimmer DP (2003). Escherichia coli spotted double-strand DNA microarrays: RNA extraction, labeling, hybridization, quality control, and data management. Methods Mol Biol.

[B17] Hayes CS, Sauer RT (2003). Toxin-antitoxin pairs in bacteria: killers or stress regulators?. Cell.

[B18] Pedersen K, Zavialov AV, Pavlov MY, Elf J, Gerdes K, Ehrenberg M (2003). The bacterial toxin RelE displays codon-specific cleavage of mRNAs in the ribosomal A site. Cell.

[B19] Christensen SK, Gerdes K (2003). RelE toxins from bacteria and Archaea cleave mRNAs on translating ribosomes, which are rescued by tmRNA. Mol Microbiol.

[B20] Zhang Y, Zhang J, Hoeflich KP, Ikura M, Qing G, Inouye M (2003). MazF cleaves cellular mRNAs specifically at ACA to block protein synthesis in Escherichia coli. Mol Cell.

[B21] Reed JL, Vo TD, Schilling CH, Palsson BO (2003). An expanded genome-scale model of Escherichia coli K-12 (iJR904 GSM/GPR). Genome Biol.

[B22] Ren D, Bedzyk LA, Thomas SM, Ye RW, Wood TK (2004). Gene expression in Escherichia coli biofilms. Appl Microbiol Biotechnol.

[B23] Gonzalez Barrios AF, Zuo R, Hashimoto Y, Yang L, Bentley WE, Wood TK (2006). Autoinducer 2 controls biofilm formation in Escherichia coli through a novel motility quorum-sensing regulator (MqsR, B3022). J Bacteriol.

[B24] Tian QB, Hayashi T, Murata T, Terawaki Y (1996). Gene product identification and promoter analysis of hig locus of plasmid Rts1. Biochem Biophys Res Commun.

[B25] Tian QB, Ohnishi M, Tabuchi A, Terawaki Y (1996). A new plasmid-encoded proteic killer gene system: cloning, sequencing, and analyzing hig locus of plasmid Rts1. Biochem Biophys Res Commun.

[B26] Falla TJ, Chopra I (1998). Joint tolerance to beta-lactam and fluoroquinolone antibiotics in Escherichia coli results from overexpression of hipA. Antimicrob Agents Chemother.

[B27] Tran JH, Jacoby GA (2002). Mechanism of plasmid-mediated quinolone resistance. Proc Natl Acad Sci U S A.

[B28] Tran JH, Jacoby GA, Hooper DC (2005). Interaction of the plasmid-encoded quinolone resistance protein QnrA with Escherichia coli topoisomerase IV. Antimicrob Agents Chemother.

[B29] Hegde SS, Vetting MW, Roderick SL, Mitchenall LA, Maxwell A, Takiff HE, Blanchard JS (2005). A fluoroquinolone resistance protein from Mycobacterium tuberculosis that mimics DNA. Science.

[B30] Brown JM, Shaw KJ (2003). A novel family of Escherichia coli toxin-antitoxin gene pairs. J Bacteriol.

[B31] Arcus VL, Rainey PB, Turner SJ (2005). The PIN-domain toxin-antitoxin array in mycobacteria. Trends Microbiol.

[B32] Pandey DP, Gerdes K (2005). Toxin-antitoxin loci are highly abundant in free-living but lost from host-associated prokaryotes. Nucleic Acids Res.

[B33] Li XZ, Nikaido H, Poole K (1995). Role of mexA-mexB-oprM in antibiotic efflux in Pseudomonas aeruginosa. Antimicrob Agents Chemother.

[B34] Lewis K LO, Wax R (2002). Drug Efflux. Bacterial Resistance to Antimicrobials: Mechanisms, Genetics, Medical Practice and Public Health.

[B35] Baba TATOYHMTYBMOTTMWBMH Systematic construction of single gene deletion mutants in Escherichia coli K-12. Manuscript in preparation.

[B36] Guzman LM, Belin D, Carson MJ, Beckwith J (1995). Tight regulation, modulation, and high-level expression by vectors containing the arabinose PBAD promoter. J Bacteriol.

[B37] Ojangu EL, Tover A, Teras R, Kivisaar M (2000). Effects of combination of different -10 hexamers and downstream sequences on stationary-phase-specific sigma factor sigma(S)-dependent transcription in Pseudomonas putida. J Bacteriol.

[B38] Martinez-Vaz BM, Xie Y, Pan W, Khodursky AB (2005). Genome-wide localization of mobile elements: experimental, statistical and biological considerations. BMC Genomics.

[B39] Khodursky AB, Peter BJ, Schmid MB, DeRisi J, Botstein D, Brown PO, Cozzarelli NR (2000). Analysis of topoisomerase function in bacterial replication fork movement: use of DNA microarrays. Proc Natl Acad Sci U S A.

[B40] Quackenbush J (2002). Microarray data normalization and transformation. Nat Genet.

[B41] Serres MH, Goswami S, Riley M (2004). GenProtEC: an updated and improved analysis of functions of Escherichia coli K-12 proteins. Nucleic Acids Res.

[B42] Boccazzi P, Zanzotto A, Szita N, Bhattacharya S, Jensen KF, Sinskey AJ (2005). Gene expression analysis of Escherichia coli grown in miniaturized bioreactor platforms for high-throughput analysis of growth and genomic data. Appl Microbiol Biotechnol.

[B43] Yang IV, Chen E, Hasseman JP, Liang W, Frank BC, Wang S, Sharov V, Saeed AI, White J, Li J, Lee NH, Yeatman TJ, Quackenbush J (2002). Within the fold: assessing differential expression measures and reproducibility in microarray assays. Genome Biol.

